# What factors impact on the implementation of clubfoot treatment services in low and middle-income countries?: a narrative synthesis of existing qualitative studies

**DOI:** 10.1186/s12891-018-1984-z

**Published:** 2018-03-02

**Authors:** Sarah Drew, Rachael Gooberman-Hill, Christopher Lavy

**Affiliations:** 1 Oxford NIHR Musculoskeletal Biomedical Research Unit, Nuffield Department of Orthopaedics, Rheumatology and Musculoskeletal Sciences, University of Oxford, Windmill Road, Headington, Oxford, OX3 7LD UK; 2 School of Clinical Sciences, University of Bristol, Learning and Research Building, Level 1, Southmead Hospital, Bristol, BS10 5NB UK; 3 National Institute for Health Research Bristol Biomedical Research Centre, University of Bristol, Bristol, UK

**Keywords:** Clubfoot, Ponseti, Narrative synthesis, Qualitative

## Abstract

**Background:**

Around 100,000 children are born annually with clubfoot worldwide and 80% live in low and middle-income counties (LMICs). Clubfoot is a condition in which children are born with one or both feet twisted inwards and if untreated it can limit participation in everyday life. Clubfoot can be corrected through staged manipulation of the limbs using the Ponseti method. Despite its efficacy and apparent availability, previous research has identified a number of challenges to service implementation. The aim of this study was to synthesise these findings to explore factors that impact on the implementation of clubfoot services in LMICs and strategies to address them. Understanding these may help practitioners in other settings develop more effective services.

**Methods:**

Five databases were searched and articles screened using six criteria. Articles were appraised using the Critical Appraisal Skills Programme (CASP) checklist. 11 studies were identified for inclusion. A thematic analysis was conducted.

**Results:**

Thematic analysis of the included studies showed that a lack of access to resources was a challenge including a lack of casting materials and abduction braces. Difficulties within the working environment included limited space and a need to share treatment space with other clinics. A shortage of healthcare professionals was a concern and participants thought that there was a lack of time to deliver treatment. This was exacerbated by the competing demands on clinicians. Lack of training was seen to impact on standards, including the nurses and midwives attending to the child at birth that were failing to diagnose the condition. Financial constraints were seen to underlie many of these problems. Some participants identified failures in communication and cooperation within the healthcare system such as a lack of awareness of clinics. Strategies to address these issues included means of increasing resource availability and the delivery of targeted training. The use of non-governmental organisations to provide financial support and methods to disseminate best practice were discussed.

**Conclusions:**

This study identified factors that impact on the implementation of clubfoot services in LMIC settings.Findings may be used to improve service delivery.

## Background

Around 100,000 children are born annually with clubfoot worldwide and of these, 80% live in middle-income counties (LMICs) [1]. Clubfoot is a condition in which children are born with one or both feet twisted inwards. Although it is not initially painful, if left untreated or ‘neglected’, clubfoot can significantly impair function and in some cases, lead to exclusion in the community [2]. Clubfoot can be corrected through staged manipulation of the limbs with plaster of Paris, a technique known as the Ponseti method. In this, approximately five manipulations are carried out on a weekly basis. This is then followed by a tenotomy, a small operation under local anesthetic to release a tight Achilles tendon. A final cast is then applied for two weeks. Correction is then maintained using foot abduction braces which must be worn full time for 2–3 months, and then nightly for 3–4 years [3]. The Ponseti technique is one of the most widely used methods to treat clubfoot throughout the world [4–6]. High numbers of treatment centers exist in LMICs [7].

Despite the efficacy of the Ponseti method and its apparent availability, previous research has identified a number of challenges to eradicating clubfoot. It has been suggested that a high proportion of children in LMICs fail to present at clinics and of those that do, many do not complete treatment [8–10]. Reasons for this have been explored in a recent meta-synthesis exploring treatment seeking behavior for clubfoot in LMICs [11]. Research has also highlighted a number of challenges to service delivery, including a lack of resources [9, 12, 13] and insufficient training for service providers [8, 12, 14]. Understanding factors that impact on the implementation of clubfoot treatment may help practitioners in other settings develop more effective services.

Qualitative studies enable researchers to explore views and experiences of service delivery [15]. Furthermore, it is thought that synthesising related qualitative studies provides findings that are relevant across multiple settings [16]. A growing body of literature aims to explore factors that impact on the implementation of clubfoot services across a range of LMICs. This literature contains findings that may relevant to providers working in similar settings.

A number of methods have been developed to synthesise qualitative research studies [17, 18]. Narrative synthesis has been chosen here to enable us to integrate findings and combine studies using a range of methodological approaches [18]. Narrative syntheses can be distinguished from traditional, narrative reviews by their systematic approach to study identification, quality appraisal and transparent methods of synthesis [18].

The aim of this study is to use a narrative synthesis to synthesise findings from existing qualitative research to explore factors that impact on the implementation of clubfoot services in LMICs and strategies to address them. This is intended to complement existing work that explores factors that impact on patient access to services across a range of LMICs, along with strategies to address them [11]. It is hoped that this will provide further information to healthcare professionals about how to develop services to better meet the needs of patients.

## Methods

A narrative synthesis was conducted in four stages: identifying studies for inclusion, appraising quality, data extraction and synthesis and reporting findings. Methods correspond to our previous meta-synthesis exploring treatment-seeking behaviour for clubfoot [11].

### Identifying studies for inclusion

Articles were identified by searching five databases: Ovid MEDLINE, PsycINFO, Embase, Global Health and CINAHL. Search criteria were broad in order to capture the highest number of publications. A combination of keyword searches and thesaurus terms or subject headings (Table 1) were applied to each database. A search filter to identify qualitative studies was then applied [19]. Databases were searched in December 2016.

**Table 1:** Search terms to explore factors that impact on the implementation of clubfoot treatment services

Patient	“Idiopathic clubfoot”	Clubfoot	“Club-foot”	Equinovarus	Talipes		
Service or intervention	Ponseti	Correction	Treatment	Tenotomy	Therapy	Service*	Surg*/Surgic*

Articles were manually screened to identify studies which fulfilled the following criteria:

1. The study related only to clubfoot services or treatments
2. The population was healthcare professionals involved in the organisation or delivery of clubfoot treatment
3. The study was conducted in a low or middle income country as defined by the World Bank [20]
4. The study explored factors that impacted on the implementation of services
5. The study was published in the last 10 years
6. Issues were explored using qualitative research methods
7. The research output was either an article or a report

Additional records were identified by searching the bibliographies of relevant articles.

### Appraising quality

To appraise study quality, the Critical Appraisal Skills Programme (CASP), a 10 point tool to help guide the evaluation of qualitative studies was used [21]. As in our previous review, items of the CASP framework were grouped into three domains: 1) study’s aims and appropriateness of methodology (items 1 and 2); 2) study design and conduct, including research design, recruitment, data collection, relationship with researcher, ethics and analysis (items 3–8); and 3) clarity of findings and value of the research (items 9–10). Each study was evaluated in relation to the three domains. Articles were then categorised into three groups. These were: ‘fully addresses CASP items’, ‘mainly addresses CASP items’ or ‘partially addresses CASP items’ [11].

Articles were independently appraised by two members of the study team (SD and RGH) andthr same judgements were reached about each article. Of these, one study was deemed to fully address CASP items, six mainly address items and four partially address items. Nevertheless all studies were included in the final review since it was thought they all provided insights into the implementation of services [22]. Table 2 details the characteristics of each study in relation to the three CASP groups and the extent to which we felt they addressed CASP items.

**Table 2:** Summary of the characteristics of papers included in the review

First author	Aims	Country	Methods	Characteristics in relation to three CASP domains	Extent it addresses CASP items
Aktintayo, O. A., 2012 [1]	To explore the dissemination of the Ponseti method, inlcuding barriers and facilitators to its implementation.	Nigeria	Semi-structured interviews and focus groups with 25 healthcare providers practising the method, 6 newly trained practitioners, 42 parents of children with clubfoot	1) Aims and appropriateness Aims of the research clearly explained. 2) Design and conduct Triangulation used such that semi-structured interviews and focus groups were used to strengthen findings. Ethical considerations were not outlined. Little information given about how thematic analysis was conducted. 3) Clarity and value Grouping of themes provided clarity to presentation. Srengths and weakenesses were not discussed. Article makes clear how findings may be applied in the future.	Partially
Boardman, A., 2011 [2]	To explore the implementation of the Ponseti method, including barriers and facilitators.	Chile, Peru, Guatemala	Semi-structured interviews with 30 healthcare providers practising the Ponseti method.	1) Aims and appropriateness Aims of research clearly outlined. 2) Design and conduct Methods appropriate and ethical issues outlined. 3) Clarity and value Ambiguity in presentation of findings since primary data and author interpretations are integrated. Relationship of study to existing literature is discussed, along with the potential application of the research.	Mainly
Gadhok, K., 2012 [3]	To explore the implementation of Ponseti method, including barriers and facilitators.	India	Semi-structured interviews with 15 orthopaedic surgeons practising Ponseti method and 15 guardians of children receiving treatment. [As part of a mixed methods study]	1) Aims and appropriateness Objectives of research clearly outlined. 2) Design and conduct Range of methods used to strengthen findings., although it is unclear what each contributed. There is no description of how patients were sampled. Reasons for study setting are clea. Ethical issues have been discussed. There is ambiguity in how data analysis has been conducted. 3) Clarity and value Study findings are clear. The presentation of findings under dominant themes and outlined in a table contributed to this. Strengths and weaknesses are considered. However, there is a lack of discussion about how the work may be used to inform practice.	Mainly
Jayawardena, A., 2013 [4]	To explore the implementation of a ‘Train the Trainer’ approach to educating practitioners about the Ponseti method.	Sri Lanka	Interviews, focus groups and observations with 162 patients and healthcare providers involved with clubfoot care.	1) Aims and appropriateness Study aims clearly outlined. 2) Design and conduct Methods used appropriate for addressing study aims. No discussion of why participants or study setting were selected. Methods of data collection discussed in detail, along with ethical considerations. However, it is unclear why the study was exempt from ethics review. Methods of analysis are not fully outlined. 3) Clarity and value Study findings are clear. The presentation of findings under dominant themes and outlined in a table contributes to this. Strengths and weaknesses are considered. However, there is a lack of discussion about how work may be used to inform practice.	Mainly
Jayawardena, A., 2011 [5]	To explore the implementation of low bandwidth webconferencing to educate practitioners about the Ponseti method.	Guatemala, Peru and Chile	Semi-structured interviews and observations with 33 healthcare providers participating in webconferencing sessions.	1) Aims and appropriateness Study aims clear. 2) Design and conduct Methods used appropriate for addressing study aims although there is no discussion of why participants or the study setting were selected. Processes of collecting observational data are not described. Ethical issues are discussed and details of the ethical review board provided. Methods of analysis are clearly outlined. 3) Clarity and value Organisation of methods into key themes provides clarity. However, there is no discussion on strengths and weaknesses or how study contributes to existing literature. Areas of future research are not outlined.	Mainly
Kingau, N. W., 2015 [6]	To explore the implementation of the Ponseti method, including those faced by guardians and healthcare professionals.	Kenya	Semi-structured interviews with 10 service providers and 10 guardians involved in clubfoot care.	1) Aims and appropriateness Study aims clear. 2) Design and conduct Methods are dsecribed in detail including how data were collected and data saturation is discussed. Ethical considerations are not outlined. Data analysis is described in detail 3) Clarity and value Themes are clear and primary data is used to enhanced the presentation of findings. There is a consideration of strengths and weaknesses. The contribution of study to exsiting literature is discussed, along with a consideration of its potential application.	Fully
Lu, N., 2010 [7]	To explore the implementation of the Ponesti method, including the experiences of guardians and healthcare providers.	China	Semi-structured interviews and focus groups with 39 healthcare providers practising the Ponseti method and 8 sets of parents of children receiving Ponseti treatment.	1) Aims and appropriateness Study aims clearly outlined. 2) Design and conduct Methods appropriate to aims and objectives of the research. Reasons for selecting participants outlined, although there is no justification for research setting. Ethical issues are discussed, including informed consent and the ethical review board. Methods of data analysis have been outlined and independent coding has been undertaken by two members of the study team to enhance confidence in the findings. 3) Clarity and value Presentation of study findings is clear. There is no consideration of strengths and weaknesses. The potential application of findings is discussed.	Mainly
Nogueira, M. P., 2013 [8]	To evaluate barriers to bracing compliance.	Brazil	Semi-structured interviews with 45 orthopaedists delivering the Ponseti method.	1) Aims and appropriateness Study aims clearly outlined. 2) Design and conduct No justification of sampling strategy or why study setting was selected. Processes of data collection and analysis not discussed. 3) Clarity and value Study findings clearly outlined and weaknesses of study design considered. How the study contributes to wider literature has not been discussed but suggestions for service improvements based on study findings are presented.	Partially
Owen, R. M., 2012 [9]	Evaluation of implementation of 10 clubfoot treatment progammes.	Democratic Republic Congo, Rwanda, Dominican Republic, Haiti, Honduras, Ethiopia, Laos, Malawi, Nepal, Paraguay, Tanzania, Zambia	Semi-structured interviews and observations of clinics with 10 clubfoot programme coordinators, 7 programme planners, regional coordinators or trainers, 10 sets of parents attending clinics and 10 trained practitioners in Ethiopia or Laos. [As part of a mixed methods study]	1) Aims and appropriateness Clear statement of study aims. 2) Design and conduct Research methods appropriate for addressing research aims. Justification for choosing semi-structured interviews and advantages of triangulation of data included. However, unclear why Ethiopia and Laos were selected for observations. No discussion of the strategy used to recruit participants. Lack of detail on how interviews and observations carried out. Ethical considerations have been dicussed including anonymisation, data storage and informed consent. Processes of data analysis clearly outlined. 3) Clarity and value Findings clear and discussion included about the strengths and limitations of the study. Researcher discusses value of research and transferability of findings to other settings.	Mainly
Palma, M., 2013 [10]	To explore barriers to the implementation of the Ponseti method.	Peru	Semi-structured interviews with 25 healthcare providers practising the Ponseti method.	1) Aims and appropriateness Aims of study are clearly outlined in the main body of the text although they are not as clear in the abstract. 2) Design and conduct Methods of data collection are appropriate although the sampling strategy and justification of study setting are not discussed. Ethical issues are outlined including the ethical review board and anonymisation of participants. There is a lack of detail about processes of data analysis. 3) Clarity and value Presentation of findings is clear and divided into themes. Strengths and weaknesses are not discussed althoug the potential application of research findings is considered.	Partially
Wu, V., 2012 [11]	To explore the impact of the Ponseti method and challenges to its implementation, including the use of web-conferencing to educate practitioners.	Vietnam	Semi-structured interviews, focus groups and observations with 12 healthcare providers delivering Ponseti treatment and 99 parents of children with clubfoot and their extended family. [As part of a mixed methods study]	1) Aims and appropriateness Aims of research clearly outlined. 2) Design and conduct Methods chosen are appropriate for addressing research questions. Use of multiple methods of data collection to increase confidence in study findings discussed. Sampling strategy outlined although reasons for selecting participants or the choice of study setting are not discussed. Processes of data collection are not outlined. There is no discussion of ethical issues. 3) Clarity and value Presentation of findings clear and strengths and weaknesses discussed. There is a consideration of how findings may be used to inform service delivery, including a series of recommendations.	Partially

### Data extraction and synthesis

Articles were imported into NVivo qualitative analysis software [23] and analysed using an inductive thematic analysis. That is, by identifying themes and subthemes in the articles [24]. Analysis was limited to the secondary interpretations of authors on account of the lack of primary data included.

### Reporting findings

The review was presented in accordance with ENTREQ guidelines, a 21 item list to improve the transparency of qualitative syntheses [16].

## Results

**Fig. 1:**
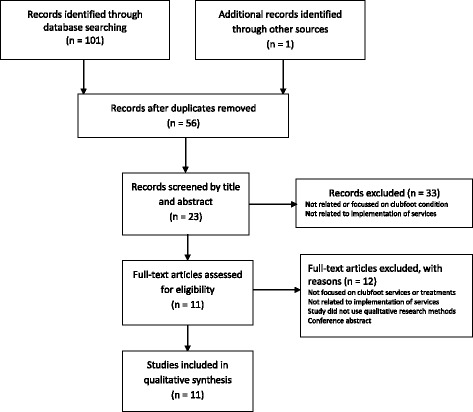
PRISMA flow chart detailing process of identifying studies relevant for inclusion

One hundred one articles were initially identified from the search criteria and 11 included in the review. The process of identifying studies for inclusion is detailed in a PRISMA flow chart in Fig. 1.

Of the 22 included studies, four were part of a mixed methods study. Studies explored the implementation of clubfoot treatment settings in the following care settings: Nigeria (1), Latin America (2), India (1), Sri Lanka (1), Kenya (1), China (1), Brazil (1), Peru (1) and Vietnam (1). One study explored implementation across 12 LMICs (Democratic Republic Congo, Rwanda, Dominican Republic, Haiti, Honduras, Ethiopia, Laos, Malawi, Nepal, Paraguay, Tanzania, Zambia). A summary of the characteristics of the studies are presented in Table 2. This includes the aims, country, methods, characteristics in relation to the three CASP domains and extent it addresses the CASP items.

Below we explore factors impacting on the implementation of clubfoot services in LMICs. We also identify strategies to address these challenges.

### Resources and working space

Across the studies, a lack of access to resources was identified as a barrier to service delivery, including a lack of casting materials [14, 25, 26] and poor quality materials [26, 27]. Acquiring abduction braces was difficult for some [8, 26–28] and healthcare professionals in Brazil were concerned that there were a lack of stores that could manufacture them to the required standard [8]. There were also challenges with the working environment and in some settings practitioners felt they had limited space to work [13, 27, 28]. In Vietnam, clinics had to share the space with patients with different conditions and the clinics were considered to provide an unpleasant environment to work due to a lack of air conditioning and overcrowding [13]. Others in Sri Lanka were concerned that they could not access a sterile environment for performing tenotomies [27].

### Staffing levels

Participants felt that there was a shortage of healthcare personnel to treat patients [28, 29]. They also thought that there was a lack of time available for delivery of treatment in clinics [9, 13, 27, 28]. Conflicting demands on healthcare professionals such as dealing with trauma cases exacerbated this problem [25, 27, 28]. In two settings children were treated in general orthopaedic clinics rather than organised clubfoot clinics. This meant they had to compete with other patients for treatment time [25, 28].

### Training and education

Participants felt there was a lack of training and education about the treatment amongst healthcare professionals [8, 9, 12–14, 25, 26, 28, 29]. It was felt that the nurses and midwives attending to the child at birth and in the early years were failing to diagnose the condition or were unaware about the existence of the method. As a result referrals were missed and treatment was delayed [12, 13, 26, 28, 29]. Some physicians were concerned that their training had lapsed since they had seen very few cases and that they were no longer equipped to deliver the treatment effectively [9, 12–14, 25]. Senior members of staff in two settings were keen to delegate more responsibilities to lab technicians and nurses but were unable to do so due to the lack of availability of training [13, 28]. In a number of settings it was found that professionals were departing from Ponseti protocols, rendering the method less or even ineffective [8, 9, 12–14, 27, 29]. This included combining the Ponseti method with massaging of the limbs [9, 13], departing from casting protocols [9, 13, 27] and prescribing abduction braces for insufficient lengths of time [8].

### Financial constraints

Underlying many of these problems were the financial constraints of the hospitals and by implication, the healthcare systems [9, 12, 25, 26, 28]. In Nigeria and Latin America, practitioners who attended the Ponseti training course had to pay for it themselves [14, 28]. To compound this difficulty, in some settings hospitals and orthopaedic surgeons received higher reimbursements for performing surgery rather than using the Ponseti treatment, discouraging the uptake of the method [9, 12, 13].

### Communication and cooperation

Some participants identified failures in communication and cooperation within the healthcare system [12, 13, 25, 28, 29]. Failures to identify the condition at birth, as identified above, meant referrals were delayed [28]. In Kenya guardians were sometimes referred to practitioners without experience in the method [29]. Referrers were often unaware of nearby Ponseti clinics, meaning carers faced unnecessarily long journeys to treatment centres, a potential barrier to treatment seeking [13]. Inter-disciplinary disagreements also hindered treatment delivery [26, 28], as did a lack of local leadership [26]. In Africa where services were delivered in collaboration with non-governmental organisations (NGOs), the level of support from the Ministry of Health was variable. Where support was low, programmes tended to develop more slowly [26].

### Lack of difficulty in delivering services

A small number of participants in China did not experience difficulties [12] and there were high levels of support amongst professionals for the method in Vietnam [13].

### Strategies to address issues identified

Strategies were suggested to address some of the issues identified. To increase the availability of abduction braces, participants in Kenya thought that local cobblers could make them to help manage costs [28]. It was also felt that training in the method should be made more widely available [13, 14, 26, 28], including a desire to train midwives and nurses in the community to identify clubfoot [13, 26, 28, 29], train more physicians [26, 28] and introduce refresher courses for those that lacked confidence in delivering the treatment [28]. ‘Hands on’ sessions, rather than ones that were more theoretical in orientation, were considered important [13]. Introducing specialised clinics or training those most likely to practice the method rather than wide-scale training programmes, was suggested as a means of ensuring high standards of treatment [9, 12, 14]. Educating support staff such as nurses and lab technicians in specific aspects of treatment was viewed as a way of ‘freeing up’ more highly qualified members of staff, enabling them to treat more children [9]. Some also felt that working with the Ministry of Health to introduce the Ponseti method into the medical curricula could facilitate its dissemination [12–14, 28]. National protocols for treatment may also help to deliver standardised care [13, 26]. Building strong and effective partnerships between NGOs and Ministries of Health was advocated [26].

In order to address the lack of funding available, it was suggested that NGOs could be used to provide support [26, 28]. There was a recognition that communication between practitioners working within the same country should be improved [13, 14]. It was also suggested that communication with other countries could also be facilitated as a means of sharing best practice. Suggested strategies for this were through virtual clinics, meetings and international conferences [13, 30]. Practitioners felt that this would give them the opportunity to discuss difficult cases, patient follow-up and experiences of establishing clinics [30]. However, the use of these was constrained by a lack of access to equipment,the internet, funding and time to attend these opportunities [30].

## Discussion

The study has identified a range of factors that impact on the successful implementation of clubfoot treatment services in LMIC settings. These were focussed on five areas: resources and working space, staffing levels, training and education, financial constraints and communication and cooperation between healthcare professionals. A lack of access to resources was identified as a challenge across the majority of settings. Difficulties with the working environment included limited space, unpleasant working conditions and a need to share treatment space with other clinics. A shortage of healthcare professionals was also a major concern, along with competing demands on clinicians. Lack of training and education was seen to impact on standards of service delivery. There was a desire for more training for midwives and nurses attending the children at birth to help aid identification, as well as those delivering treatment. Financial constraints were seen to underlie many of these problems. Only a small number of participants in China did not experience any difficulties. Strategies to address these issues included means of increasing resource availability and delivering targeted training for healthcare professionals. The use of non-governmental organisations to provide financial support and methods to disseminate best practice were discussed. Although treatment was delivered across a range of settings, there was little difference between factors impacting on implementation or on strategies to address them. Findings also support those from a recent review that has highlighted the lack of physical resources and training for healthcare providers delivering the Ponseti method in of LMICs [31]. This study complements existing work that has explored factors that impact on patient access to clubfoot treatment, along with strategies to address them [11].

### Strengths and weaknesses

We undertook a systematic and exhaustive search to identify studies. Although we cannot say with certainty that all literature was captured, by refining search terms with the study team, including a clinician with experience of delivering these services, meant this was probably the case. Using the ENTREQ guidelines, a 21 item list for improving the transparency of qualitative syntheses [16], also enhanced the presentation of our study.

The quality of the studies identified was variable and may have limited the findings of the review. According to our CASP quality appraisal [21], one study was deemed to fully address items, six mainly address items and four partially address items. To enhance confidence in these assessments, all 11 articles were independently appraised by two members of the study team and both arrived at the same judgements. However, a decision was made to include all the articles in the synthesis since they were all seen to provide valuable information about the issues under study.

We made the decision to limit our review to published qualitative studies that were more likely to be of higher quality and provide more systematic exploration of issues than grey or unpublished literature.

### Further research

Further research is now needed to evaluate the implementation of clubfoot services in other LMIC settings, along with suggested strategies to improve service delivery. Doing so would provide additional information about how best to deliver clubfoot treatment services.

## Conclusions

This study has identified factors that impact on the implementation of clubfoot services in LMICs and strategies to address them. These findings may help professionals across a range of LMICs implement services more effectively in the future.

## Abbreviations

CASP: Critical Appraisal Skills Programme
LMICs: Low and middle-income countries
